# SphK1 confers resistance to apoptosis in gastric cancer cells by downregulating Bim via stimulating Akt/FoxO3a signaling

**DOI:** 10.3892/or.2014.3391

**Published:** 2014-08-07

**Authors:** HUAPING XIONG, JICHENG WANG, HONGYU GUAN, JUEHENG WU, RU XU, MIN WANG, XIA RONG, KE HUANG, JIETING HUANG, QIAO LIAO, YONGSHUI FU, JIE YUAN

**Affiliations:** 1 Guangzhou Blood Center, Institute of Blood Transfusion, Guangzhou, Guangdong 510095, P.R. China; 2 Guangdong Women’s and Children’s Hospital, Medical Genetics Center, Guangzhou, Guangdong 510010, P.R. China; 3 Key Laboratory of Tropical Disease Control, Ministry of Education, Sun Yat-Sen University, Guangzhou, Guangdong 510080, P.R. China; 4 Department of Endocrinology and Diabetes Center, The First Affiliated Hospital of Sun Yat-Sen University, Guangzhou, Guangdong 510080, P.R. China

**Keywords:** sphingosine kinase 1, gastric cancer, apoptosis, forkhead box O3a

## Abstract

We previously reported that sphingosine kinase 1 (SphK1), an enzyme that catalyzes the production of sphingosine- 1-phosphate (SIP), is upregulated in human gastric cancer and predicts poor clinical outcome. In the present study, we used known differential effects of UV irradiation on human MGC-803 gastric cancer cells to determine their effect on SphK1 activity. Ectopic expression of SphK1 in MGC-803 gastric cancer cells markedly enhanced their resistance to UV irradiation, whereas silencing endogenous SphK1 with shRNAs weakened this ability. Furthermore, these antiapoptotic effects were significantly associated with decrease of Bim, an apoptosis-related protein. We further demonstrated that SphK1 could downregulate the transcriptional activity of forkhead box O3a (FoxO3a) by inducing its phosphorylation, which was found to be associated with the PI3K/Akt signaling. Taken together, our study supports the theory that SphK1 confers resistance to apoptosis in gastric cancer cells via the Akt/FoxO3a/Bim pathway.

## Introduction

Gastric cancer has the second highest incidence and mortality rates in the world, and the highest in China (1,2). In terms of treatment, surgery remains the only effective treatment for gastric cancer in resectable stages. However, approximately 84% of patients with gastric cancer have advanced disease (3). Therefore, various adjuvant chemotherapy and radiotherapy protocols have been compared with surgery alone in advanced gastric cancer, but the 5-year survival analysis suggested only a moderate improvement following adjuvant treatments (4).

In our previous study, poor prognosis of patients with gastric cancer was found to be correlated with elevated expression of sphingosine kinase 1 (SphK1), one of the SphK isoenzymes that generate the bioactive lipid mediator, sphingosine-1-phosphate (S1P) (5). The S1P, sphingosine (SPH), and sphingolipid metabolite ceramide (Cer) play key roles in the determination of various cellular functions, including cell proliferation, survival and mortality. S1P is involved in stimulating growth and suppressing apoptosis, and, in contrast, Cer and SPH inhibit proliferation and promote apoptosis. Therefore, the relevance of S1P, Cer and SPH lead to a proposal of ‘sphingolipid rheostat’, which is critical for determination of cell fate (6,7). Notably, SphK, the enzymes that phosphorylate SPH to form S1P, plays a pivotal role in sphingolipid rheostat. The proliferative and anti-apoptotic messenger S1P is produced by SphK, while SphK also decreases levels of pro-apoptotic Cer and SPH (6–11). Accumulating evidence further demonstrates the anti-apoptotic effects of SphK1. For example, SphK1 could protect cancer cells against apoptosis from apoptosis induced by TNF-a, ionizing radiation or anticancer drugs, due to increased ceramide levels (12,13). Meanwhile, ectopic overexpression of SphK1 inhibited caspase cleavage and activated the pro-apoptotic kinase JNK to inhibit PC12 cells from apoptosis caused by growth factor withdrawal or exogenous Cer (14). Moreover, Bonhoure *et al* demonstrated that the resistance to doxorubicin and etoposide-induced cell death were conferred in SphK1-overexpressing HL-60 leukemia cells (15). In addition, Pchejetski *et al* found that the resistance to camptothecin or docetaxel in prostate cancer cells was also associated with elevated SphK1 activity (16). Taken together, these reports indicate that SphK1 is involved in the regulation of cancer cell apoptosis.

It has been well demonstrated that the serine/threonine kinase Akt (also known as PKB) acts as one of the most important protein kinases in various physiological and pathological conditions, particularly in cancer (17). Many oncoproteins and tumor suppressors are involved in the Akt pathway to exert their biologic function. SphK1 acts as an oncoprotein and facilitates Akt signaling activation in several human cancers, such as glioblastoma, colon cancer and erythroleukemia (18–20). Notably, several studies documented that Akt can phosphorylate forkhead box O (FoxO) proteins and the regulation of FoxO proteins is mainly due to the phosphatidylinositol 3-kinase (PI3K)-Akt signaling pathway (21). Based on these previous findings, we hypothesized that SphK1 may be involved in gastric cancer tumorigenesis via regulation of the Akt/FoxO3a signaling pathway.

In the present study, we reported the role of SphK1 in the sensitization of radiation-resistant MGC-803 gastric cancer cells to UV-induced apoptosis. We also demonstrated that the anti-apoptotic effect of SphK1 on gastric cancer cells is associated with the activation of the Akt/FOX3a pathway, suggesting that inhibition of SphK1 may represent a novel approach to the treatment of gastric cancer.

## Materials and methods

### Cell line and retroviral infection

Gastric cancer cell line MGC-803 was maintained in DMEM medium (Invitrogen, Carlsbad, CA, USA) supplemented with 10% fetal bovine serum (FBS; HyClone, Logan, UT, USA). For ectopic overexpression, an SphK1 expression construct from subcloning PCR-amplified full-length human SphK1 cDNA was inserted into the pMSCV plasmid. For depletion of SphK1, two human SphK1-targeting siRNA sequences were cloned into pSuper-retro-puro to generate pSuper-retro-SPHK1-RNAi(s), respectively, and the sequences were: RNAi#1, GGCTGAAATCTCCTTCACG; RNAi#2, GGGCAAGGCCTTGCAGCTC. Retroviral production and infection were performed as previously described (22). Stable cell lines expressing SphK1 or SphK1 shRNAs were selected for 10 days with 0.5 μg/ml puromycin.

### Western blot analysis

Western blot analyses were performed according to the standard method as described previously (23), using anti-SphK1 antibody (Abgent, San Diego, CA, USA); anti- Akt, anti-p-Akt, anti-cleaved caspase 3, anti-PARP, anti-Bim, anti-bax, anti-Bcl-xL, anti-p-FoxO3a, and anti-Bcl-2 antibodies (Cell Signaling, Danvers, MA, USA). The membranes were stripped and re-blotted with an anti-α-tubulin Ab (Sigma, St. Louis, MO, USA) as a loading control.

### RNA extraction and real-time RT-PCR

Total RNA from cultured cells was extracted using TRIzol reagent (Invitrogen) according to the manufacturer’s instructions. Real-time PCR was performed according to standard methods as described previously (23). Primer sequences were: SphK1 forward, 5′-CTTGCAGCTCTTCCGGAGTC-3′ and reverse, 5′-GCTC AGTGAGCATCAGCGTG-3′; GAPDH forward, 5′-GACTC ATGACCACAGTCCATGC-3′ and reverse, 5′-AGAGGCAG GGATGATGTTCTG-3′. Expression data were normalized to the housekeeping gene GAPDH as a loading control.

### TUNEL assay

The DeadEnd™ Fluorometric TUNEL System (Promega, Madison, WI, USA) was performed according to the manufacturer’s instructions. Briefly, after incubation for 24 h, cells were treated by UV irradiation (20 J/m^2^), and were then washed with cold PBS. Fresh 4% formaldehyde solution was used to fix for 25 min at 4°C, and the fixed slides were washed with PBS for 5 min once, followed by 0.2% Triton X-100 in PBS for 5 min. After a 5-min wash with PBS, 100 μl equilibration buffer was used to cover cells for 5 min, followed by a 60-min incubation with 2X SSC at 37°C to terminate the reaction. After washing with PBS 5 min once, the samples were stained with 1 μg/ml propidium iodide (PI) solution in the dark for 15 min, followed by 1 μg/ml DAPI solution in the dark for 15 min. After a final wash with ddH_2_O for 5 min at room temperature and air-drying, samples were immediately analyzed under a fluorescence microscope. A standard fluorescein filter was set to view the green fluorescence of fluorescein at 520 nm, the red fluorescence of PI at 620 nm, and the blue DAPI at 460 nm.

### Annexin V binding assay

The ApopNexin™ FITC Apoptosis Detection Kit (Millipore, Lake Placid, NY, USA) was performed to quantitate apoptotic cells, according to the manufacturer’s instructions. Briefly, cells were treated with UV irradiation (20 J/m^2^) followed by 6 h incubation. After a 5-min wash with PBS, 150 μl of an Annexin V antibody in binding buffer was added with incubation for 15 min at room temperature, followed by addition of 1.5 μl of PI at 1 mg/ml and a further incubation for 5 min. Subsequently, after washing with the Annexin V Binding Buffer, samples were immediately analyzed under a fluorescence microscope equipped with a filter for fluorescein isothiocyanate (excitation, 490 nm; emission, 525 nm), and PI staining was assessed with the filter for Texas red (excitation, 570 nm; emission, 610 nm).

### Measurement of S1P levels in cellular assays

An S1P competitive ELISA kit (Echelon Biosciences, Salt Lake City, UT, USA) was used to analyze S1P level according to the manufacturer’s instructions. In brief, cells were serum-starved for 24 h and lysed in 400 μl of the lysis buffer. Diluted cell lysate (1:10 in delipidized human sera) was analyzed with the Echelon S1P ELISA using the anti-S1P antibody, and the absorbance was measured at 450 nm using a microplate reader.

### Statistical analysis

Comparisons between groups for statistical significance were performed with a two-tailed Student’s t-test. A P-value <0.05 (using a two-tailed paired t-test) was considered to indicate a statistically significantly difference.

## Results

### Overexpression and silencing of SphK1 expression in gastric cancer cells

**Figure 1:**
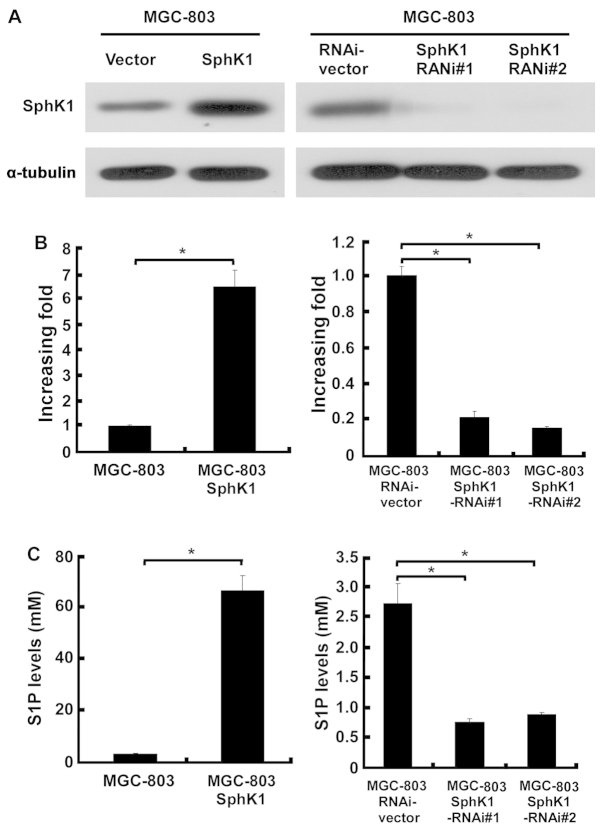
MGC-803 cell lines were constructed to stably express either SphK1 cDNA or SphK1 shRNAs. (A) Expression of SphK1 was examined in indicated cells. α-tubulin was used as a loading control. (B) Real time-PCR analysis of SphK1 mRNA expression in parental and engineered MGC-803 cells. Expression of GAPDH was used as a control of gene expression. (C) The cellular S1P levels were examined in SPHK transduced cells and SphK1-knockdown cells. Data were obtained from three independent experiments with similar results. ^*^P<0.05.

To investigate the effect of SphK1 expression in gastric cancer cells, retrovirally mediated overexpression and silencing of SphK1 were conducted in this study. In order to demonstrate the expression levels of SphK1 in the engineered MGC-803 gastric cancer cells, western blotting and real-time PCR analysis were performed. After retroviral transduction and puromycin selection, SphK1 protein and mRNA levels were markedly increased or reduced by, respectively, pMSCVSphK1 and pSuper-shSphK1, as compared to the vector-control cells (Fig. 1A and B). As it is widely known that activation of SphK1 requires post-translational steps, we examined the cellular S1P level in the above engineered MGC-803 cells, and as shown in Fig. 1C, overexpressing SphK1 starkly increased 66-fold, and knockdown of SphK1 reduced 3-fold, respectively, the cellular S1P level in MGC-803 cells as compared with the control cells.

### Sensitization of gastric cancer cells to pro-apoptotic stimuli

**Figure 2:**
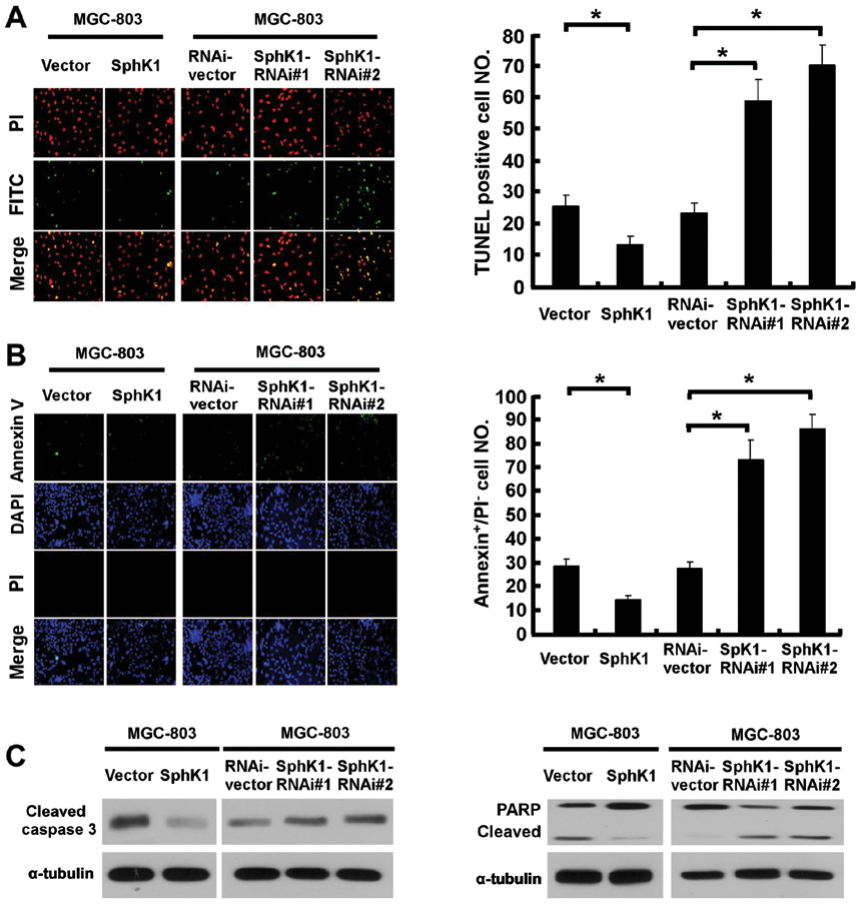
SphK1 expression prevents UV irradiation-induced cell death. (A) Representative immunofluorescent images (left panel) and quantification (right panel) of TUNEL-stained cells in indicated cells after UV irradiation (20 J/m^2^). The numbers of TUNEL-positive cells were counted from 10 random fields and are presented as percentages of total cell numbers. ^*^P<0.05. (B) Immunofluorescent images (left panel) and quantification (right panel) of Annexin V/PI staining of vector-, SphK1- or SphK1 shRNA-transduced MGC-803 cells after UV irradiation (20 J/m^2^). Numbers are cells counted from 10 random fields. ^*^P<0.05. (C) Western blotting for proteolytic cleavage of pro-caspase-3 and PARP in vector-, SphK1- or SphK1 shRNA-transduced MGC-803 cells after UV irradiation (20 J/m^2^), using α-tubulin as loading control.

We then attempted to further understand and characterize the anti-apoptotic activity of SphK1 to pro-apoptotic stimuli. As shown in Fig. 2A, the number of apoptotic cells induced by UV irradiation decreased approximately 2-fold in SphK1-overexpressing cells, and increased 3-fold in SphK1-knockdown MGC-803 cells, as compared to the control cells. Similarly, the result of Annexin V-binding assay was consistent with the TUNEL assay (Fig. 2B), suggesting that SphK1 promoted resistance of gastric cancer cells against radiation-induced apoptosis. Furthermore, the effect of SphK1 on apoptosis was associated with decreased caspase-3 and PARP activation in UV radiation-treated SphK1-overexpressing cells, and a contrary effect was observed in SphK1-knockdown cells (Fig. 2C).

### SphK1 regulates the expression of Bim to induce gastric cancer cell survival through the Akt/FoxO3a pathway in gastric cancer cells

**Figure 3:**
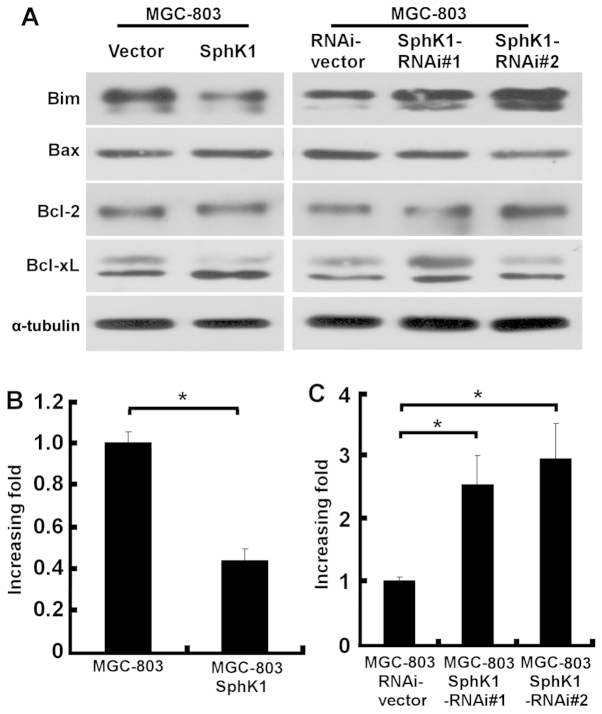
SphK1 regulates the expression of Bim. (A) Western blotting for Bim, Bax, Bcl-2 and Bcl-xL levels in indicated cells after UV irradiation (20 J/m^2^), using α-tubulin as loading control. (B and C) Real-time PCR analyses of Bim expression in indicated cells after UV irradiation (20 J/m^2^). Expression of GAPDH was used as a control of gene expression. ^*^P<0.05.

We next examined the possible involvement of signaling molecules in the effect of SphK1 on apoptosis. Notably, ectopic overexpression of SphK1 did not affect the expression of apoptosis regulators Bax, Bcl-2 and Bcl-xL, but, instead, significantly suppressed the expression of Bim (Fig. 3A). In contrast, Bim level was significantly upregulated in SphK1-knockdown gastric cancer cells (Fig. 3A), suggesting a specific regulatory role of SphK1 in cell apoptosis. To understand at which level SphK1 is involved in the regulation of Bim expression, realtime PCR was performed to examine the mRNA levels of Bim in SphK1-overexpressing, SphK1-knockdown and vector control gastric cancer cells. As exhibited in Fig. 3B, upregulation of SphK1 in MGC-803 cells was found to decrease the mRNA levels of Bim, whereas Bim mRNA was significantly elevated in SphK1-knockdown MGC-803 cells, as compared with those in vector-control cells (Fig. 3C).

**Figure 4:**
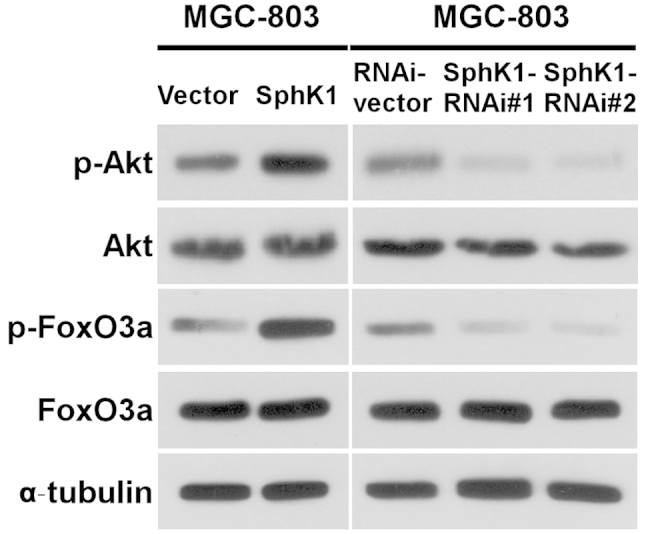
Upregulation of SphK1 activates the Akt/FoxO3a signaling. Western blot analysis for phosphorylated Akt (p-Akt), total Akt, phosphorylated FoxO3a (p-FoxO3a) and total FoxO3a levels in indicated cells, using α-tubulin as loading control.

Given that the expression of Bim could be transcriptionally regulated by FoxO3a and the transcriptional activity of FoxO3a could be modulated by Akt phosphorylation, we examined whether upregulating SphK1 expression could activate Akt/FoxO3a signaling. As shown in Fig. 4, the phosphorylation levels of Akt and FoxO3a were indeed increased in ectopically SphK1-transduced gastric cancer cells. In contrast, the expression levels of phosphorylated FoxO3a and phosphorylated Akt in SphK1-knockdown cells were decreased (Fig. 4), indicating that SphK1 regulates the expression of Bim via Akt/FoxO3a signaling.

## Discussion

Several lines of evidence have indicated that increased resistance to apoptosis is a hallmark of most types of cancer (24). Deregulation of apoptotic or pro-apoptotic pathways is one of the most important events for tumor development and progression. Moreover, the failure of treatment by chemotherapy or radiotherapy may be associated with apoptotic programming. Thus, understanding the mechanisms of apoptosis/survival process in a specific type of cancer may provide new insights into developing more effective therapeutic strategies. Mounting evidence has shown that SphK1 plays an important role in regulating tumor cell apoptosis. For example, Bonhoure *et al* found that HL-60 acute myeloid leukemia cells resist doxorubicin and etoposide-induced cell death due to SphK1 overexpression (15). Resistance to camptothecin and docetaxel in PC-3 and LNCaP prostate cancer cells, respectively, is associated with stimulation of SphK1 activity (16). Here, we demonstrated that SphK1 indeed plays an important role in the anti-apoptotic state of gastric cancer cells. Ectopic expression of SphK1 in MGC-803 cells markedly enhanced their resistance to apoptosis induced by UV irradiation, a commonly used model to study radiotherapy, whereas suppressing SphK1 expression with shRNAs markedly abrogated the ability of MGC-803 cells to resist UV-induced cell death, suggesting that SphK1 contributes to sustaining the unwanted survival of gastric cancer cells under radiotherapy.

FoxO transcription factors including FoxO1, FoxO3a, FoxO4 and FoxO6 contribute to the regulation of downstream gene expression which modulates several biologic phenomena, such as apoptosis, proliferation and DNA repair (25–27). Multiple reports have revealed that phosphorylation of FoxO proteins by Akt can promote apoptosis in cancer cells (28–30). It is of note that phosphorylation of FoxO3a triggers apoptosis through a mechanism that depends on a pro-apoptotic gene Bim that encodes a member of the BH3-only subgroup of Bcl-2 family proteins (31). Bim has been shown to be transcriptionally regulated by FoxO proteins (32,33). Furthermore, two functional FRE sites present in the Bim promoter support that FOXO transcription factors directly activate Bim gene expression and promote apoptosis (34). Consistent with these results, the present study demonstrated that the expression level of FoxO3a, an upstream regulator of Bim, is suppressed in gastric cancer cells expressing high level of SphK1, whereas it was upregulated in SphK1-knockdown gastric cancer cells. Furthermore, we showed that downregulation of FoxO3a by SphK1 is associated with Akt phosphorylation. Collectively, the present study suggests a novel signaling cascade that links SphK1 to the antiapoptotic property of cancer cells and provides novel therapeutic targets against gastric cancer. These results also warrant further investigation into how SphK1 activates the PI3K/Akt pathway, currently being carried out in our laboratory.
